# Preparation of desiccation-resistant aquatic-living *Nostoc flagelliforme* (Cyanophyceae) for potential ecological application

**DOI:** 10.1111/1751-7915.12279

**Published:** 2015-04-06

**Authors:** Xiang Gao, Yi-Wen Yang, Li-Juan Cui, De-Bao Zhou, Bao-Sheng Qiu

**Affiliations:** 1 School of Life Sciences, Hubei Key Laboratory of Genetic Regulation and Integrative Biology, Central China Normal UniversityWuhan, 430079, China; 2 School of Biological Sciences and Biotechnology, Baotou Normal CollegeBaotou, 014030, China

## Abstract

*Nostoc flagelliforme* is a terrestrial edible cyanobacterium that grows in arid and semi-arid steppes. The continued over-exploitation in the last century has led to a sharp decline of this resource and a severe deterioration of the steppe ecology. Liquid-cultured *N. flagelliforme* serves as promising algal ‘seeds’ for resource restoration. In this study, macroscopic (or visible) aquatic-living colonies (MaACs) of *N. flagelliforme* were developed under weak light and high nitrogen conditions. In a 24 day shake-flask culture, MaACs were propagated by about 4.5-fold in biomass without loss of their macro-morphology; at the same time, the addition of weak UV-B treatment resulted in slightly bigger MaACs. Polyvinylpyrrolidone (PVP) k30, a water-soluble polymer, was used to generate the coating around MaACs, and after full desiccation, the coated MaACs could recover their photosynthetic physiological activity when rehydrated, with 4% PVP k30 for coating being most effective. In contrast, PVP k30-coated microscopic aquatic-living colonies of *N. flagelliforme* and non-coated MaACs showed no resistance to full desiccation. The macroscopic morphology or structure of MaACs should be crucial for the formation of protection by PVP k30 coating. PVP k30-coated MaACs were more approaching to actual application for resource restoration.

## Introduction

*Nostoc flagelliforme* is a terrestrial, macroscopic, filamentous cyanobacterium that grows in arid and semi-arid steppes within China, Algeria, Czechoslovakia, France, Mexico, Mongolia, Morocco, Russia, Somalia and the United States (Gao, 1998). *N. flagelliforme* (colonial filament) is usually 5–60 cm long, 0.2–1 mm in diameter, in which parallel arranged rosary-like cells are encased in dense extracellular polysaccharides (EPS) sheath (Qian *et al*., 1989; Gao, 1998). As a pioneer species, *N. flagelliforme* can fix a large amount of atmospheric nitrogen and synthesize abundant EPS (Yu *et al*., 2010; Gao *et al*., 2012), which nourish the barren steppes. This species has served as a food delicacy for more than 2000 years in China and Southeast Asia (Gao, 1998); however, the continued over-exploitation during the last century in China has led to a sharp decline of this resource and a severe deterioration of the steppe ecology (Gao *et al*., 2014). The natural recovery of the *N. flagelliforme* source should be a very long-term process because the annual growth rate of this species is less than 6% in native habitats (Dai, 1992). Therefore, although *N. flagelliforme* has been prohibited from further collection and trading in China since June 2000, it remains as a challenging task to restore this source in the arid steppes.

Developing a large number of algal ‘seeds’ may be a vital solution for resource restoration. Reproduction of *N. flagelliforme* can take place in several ways (Gao, 1998). Vegetable reproduction is one of the main ways for its proliferation and spreading in native habitats. Thousands of tiny filamentous ‘seeds’ can be prepared by fragmenting air-dried natural *N. flagelliforme*. However, this means may be still limited for ecological application because to prepare enough ‘seeds’ it is still required to collect massive natural *N. flagelliforme*. The artificial cultivation of *N. flagelliforme* that can form natural colonial morphology has been attempted in solid mediums or fields during the past 30 years (Gao, 1998; Feng *et al*., 2012). However, the slow growth associated with low productivity makes it impractical for application (Gao *et al*., 2014). When natural *N. flagelliforme* is cultured under aquatic conditions, it will lose its visible morphology because of the disintegration of EPS sheath and form aquatic-living colonial filaments that are 10–30 μm wide (Gao and Ye, 2003). These microscopic aquatic-living colonies (MiACs) of *N. flagelliforme* can be rapidly and massively propagated in flasks and photobioreactors (Su *et al*., 2008; Yu *et al*., 2009; Ding *et al*., 2013). Thus, aquatic-living algal ‘seeds’ represents the promising means for ecological application. The native habitats of *N. flagelliforme* are characterized with extreme dryness, drastic temperature changes and intense solar radiation (Qian *et al*., 1989; Gao, 1998). MiACs were reported to exhibit good adaptability to solar radiation (Lai and Gao, 2009), cold (Wang *et al*., 2011), salt (Ye and Gao, 2004) and certain dehydration (Gao and Ye, 2003; Ye *et al*., 2012) stresses. However, we found that after experiencing full desiccation, MiACs of *N. flagelliforme* could not recover the photosynthetic physiological activity upon rehydration, which represents a great obstacle for their application in harsh native environments.

In this study, we reported the obtaining and shake-flask culture of macroscopic (or visible) aquatic-living colonies (MaACs) of *N. flagelliforme* in contrast to MiACs, and the improvement of the vitality of MaACs upon full desiccation. Desiccation stress-resistant MaACs should be currently the most promising algal ‘seeds’ for ecological application.

## Results and discussion

### The induced generation of MaACs from MiACs

**Figure 1:**
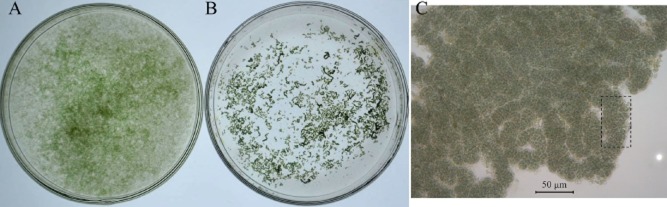
The suspensions of (A) MiACs and (B) MaACs of *N. flagelliforme* and (C) the appearance of a MaAC under microscope. Suspensions were contained in 9 cm diameter Petri dishes. The rectangle indicates a tiny colonial filament (or MiAC). A MaAC (0.2–0.5 mm in width) was morphologically composed of aggregated MiACs (10–30 μm in width). Suspensions of MiACs were prepared by culturing natural *N. flagelliforme* filaments in BG11 solution for 1–2 months according to Gao and Ye (2003); MaACs were induced from MiACs in this study. Suspensions were cultured at 25°C.

Free-living cells were released from ruptured natural colonies of *N. flagelliforme* during aquatic culture and developed into MiACs under light conditions (20–60 μmol photons m^−2^ s^−1^) (Gao and Ye, 2003). The MiACs were not visible by naked eyes, and their suspension was shown in Fig. 1A. Under ventilation culture, visible MaACs (Fig. 1B) occasionally occurred, but the proportion was quite small. Under microscope, an MaAC in fact consists of some tiny colonial filaments (or MiACs) (Fig. 1C). We had accidentally found that nearly all MiACs changed to MaACs after the former, which had been long-term statically cultured under a weak light, were transferred to fresh BG11 solution (Stanier *et al*., 1971) for 2 weeks. Light rather than temperature was crucial for the formation of filamentous colonies of MiACs (Gao and Ye, 2003). According to further attempts, we found relatively low initial concentration (e.g. below 0.01 OD_730_), high nitrogen concentration [e.g. 1–2.5 fold of nitrogen level as that (1.5 g l^−1^ NaNO_3_) in BG11 solution] and weak light (e.g. 1–5 μmol photons m^−2^ s^−1^) were beneficial to achieve more probability of success to turn a large number of MiACs into MaACs in static culture conditions. EPS extracted from aquatic-living *N. flagelliforme* have a high intrinsic viscosity (Huang *et al*., 1998). The ratio of EPS to biomass should be an important factor affecting the aggregation of colonial filaments to form MaACs. Too rapid agitation by impeller led to the more release of capsular EPS (tightly attached to cell surface) into the medium and caused the disintegration of MiACs (Su *et al*., 2008). The increase of light intensity (e.g. from 10 to 180 μmol photons m^−2^ s^−1^) led to increased biomass yield and polysaccharide production in liquid culture (Gao and Ye, 2003; Yu *et al*., 2010); however, the maximal EPS/biomass ratio was observed at 20 μmol photons m^−2^ s^−1^ (Yu *et al*., 2010). Light compensation point of aquatic-living *N. flagelliforme* was 6–16 μmol photons m^−2^ s^−1^ (Zhao *et al*., 2005; Su *et al*., 2006). At lower illumination (e.g. 3–8 μmol photons m^−2^ s^−1^), cell growth was inhibited but a higher EPS/biomass ratio was observed (Bi and Hu, 2004). Other unfavourable conditions such as cold (Wang *et al*., 2011) and UV radiation (He *et al*., 2002; Lai and Gao, 2009) also resulted in a thicker EPS sheath around *N. flagelliforme* cells (or potentially high EPS/biomass ratio), which is consistent with the crucially protective role of EPS for desert cyanobacteria in extremely changeable environments (Chen *et al*., 2009; Gao *et al*., 2012). MiACs cultured in BG11 medium had a higher EPS/biomass ratio than those in BG11_0_ (nitrogen free) medium (Bi and Hu, 2004). The increase of nitrogen nutrition in liquid suspension cultures of MiACs (e.g. from 1 to 2.5 g l^−1^ NaNO_3_) promoted increasing biomass yield coupled with a faster production of EPS, resulting in an increasing EPS/biomass ratio; at 3.5 g l^−1^ NaNO_3_, the ratio reduced (Yu *et al*., 2010). The chlorophyll *a* (chl *a*, which is commonly used as a proxy for relative biomass) and EPS contents were measured in MaACs for analysing the EPS/Chl *a* ratio (Table 1). The values of EPS/Chl *a* ratio for MaACs were much higher than those for MiACs from various culture conditions but were lower than that for natural *N. flagelliforme*. These results imply that the induced formation of MaACs under weak light and high nitrogen conditions may be correlated with the occurrence of high EPS/biomass ratio. The precise mechanisms still await further investigation because these conditions were quite important but not all for the massive induction of MaAC formation as above mentioned.

**Table 1:** The proportion of EPS to Chl *a* in MaACs, MiACs and natural *N. flagelliforme*

Samples	NaNO_3_ (g l^−1^)	Chl *a* (mg l^−1^)	EPS (mg l^−1^)	EPS/Chl *a* ratio
MaACs	1.5^a^	1.94	69.7	35.9
	3.75^a^	1.40	92.3	65.9
	1.5^b^	5.06	173.3	34.2
	3.75^b^	2.93	160.5	54.8
MiACs	1.5^c^	2.03	29.3	14.4
	3.75^c^	2.23	50.8	22.8
	1.0–3.5^d^	5.05–6.41	44–100	8.7–15.6
Natural clonies	/	/	/	81.6

**a, b.** Respectively 10 and 20 day MaACs samples, cultured in a 250 ml flask in a shaker with rotating speed of 100 r.p.m.

**c.** MiACs were cultured in static condition in a 250 ml flask for 20 days, which was formerly performed in our laboratory.

**d.** Data from Yu and colleagues (2010); cultured for 20 days in a 20 l turbine-agitated photobioreactor. The temperature for these cultures was 25°C, and light intensity was 60 μmol photons m^−2^ s^−1^. Natural colonies of *N. flagelliforme* were collected from Inner Mongolia, China, in 2012, in which Chl *a* and EPS contents were, respectively, 2.08 and 169.7 mg g^−1^ dry weight. Chl *a* was extracted from the samples with 95% ethanol and determined as described by Gao and colleagues (2014). Polysaccharides were extracted with hot water (Huang *et al*., 1998) and determined as described by Su and colleagues (2008).

### Massive production of MaACs in shaking culture

**Figure 2:**
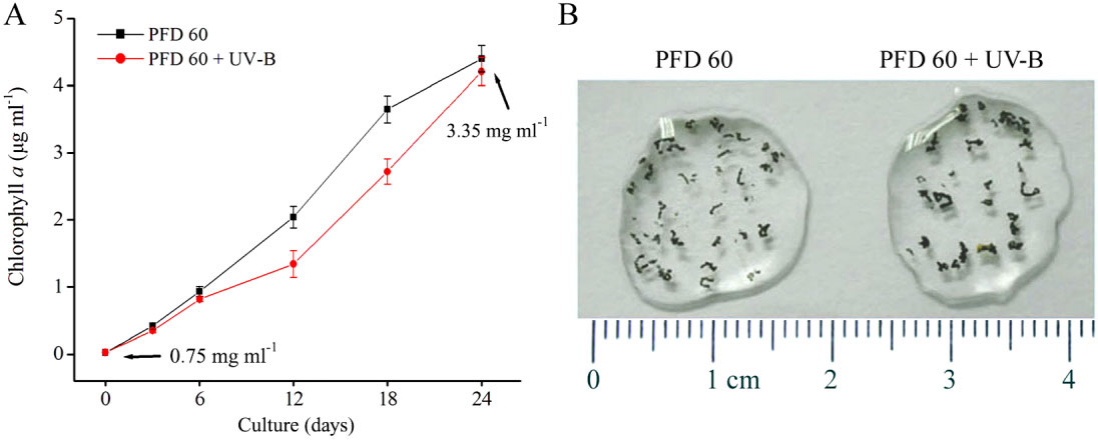
The (A) propagation and (B) morphology of MaACs under shaking cultures. Suspensions were cultured in 500 ml flask containing BG11 solution supplemented with extra 2.25 g l^−1^ NaNO_3_ at 25°C. The shaking velocity was 100 r.p.m. PFD 60, continuous white light with a PFD of 60 μmol photons m^−2^ s^−1^; PFD 60+ UV-B, the continuous white light coupled with periodic UV-B of 0.2 W m^−2^ (9 h light/15 h dark). Data represented as the mean ± SD (*n* = 3). The initial and final biomasses indicated in A refer to dry weight. The UV-B treated MaACs (0.33 ± 0.10 mm wide, *n* = 30) were significantly bigger than non-treated MaACs (0.24 ± 0.08 mm wide, *n* = 30) (Student's *t*-test, *P* < 0.05).

The massive production of MaACs as algal ‘seeds’ was of great significance for ecological application. The ideal culture conditions for fast production of aquatic-living *N. flagelliforme* were formerly reported (Su *et al*., 2008; Yu *et al*., 2009; 2010). Under low UV-B radiation, single trichomes with 50–200 vegetative cells could develop into macroscopic filamentous thalli (Feng *et al*., 2012). Accordingly, we attempted to culture MaACs in a shaker under two conditions: white fluorescent lights with a continuous photon flux density (PFD) of 60 μmol photons m^−2^ s^−1^ (PFD 60); and a continuous PFD 60 plus a 0.2 W m^−2^ UV-B treatment. Chl *a* concentration and dry weight were measured during a 24 day culture (Fig. 2A). Under both conditions, MaACs were rapidly propagated, with a slightly lower growth for the latter condition; nevertheless, they reached similar amounts of cell concentration at 24 days. During this process, the MaACs achieved a ∼ 4.5 fold of biomass increase, from an initial 0.75 mg ml^−1^ to a final 3.35 mg ml^−1^ in dry weight. Moderate UV-B radiation results in oxidative stress and damage (He *et al*., 2002). However, either weak (e.g. 0.1 W m^−2^) or higher (e.g. 1–5 W m^−2^) UV-B radiation could induce *N. flagelliforme* cells to produce more EPS and UV-screening compounds, scytonemin and mycosporine-like amino acids (MAAs) (Ferroni *et al*., 2010; Feng *et al*., 2012; Yu and Liu, 2013). The protective effect of scytonemin and MAAs has been demonstrated in a wide range of organisms (Ferroni *et al*., 2010). UV-B radiation also gave rise to the increase of carotenoid content and superoxide dismutase and catalase activities in MiACs (Lai and Gao, 2009; Sa *et al*., 2013). The combination of these protective factors may aid in the recovery of rapid growth of UV-B treated MaACs in the later phase. More importantly, the increase of UV-protective compounds in MaACs with UV-B treatment should be beneficial for the colonies to survive solar radiation in natural habitats. Notably, the overall morphology of visible MaACs was relatively stable during the shake-flask culture, with the UV-B treated MaACs being slightly bigger than non-treated MaACs (Fig. 2B). In addition, we observed that these MaACs did not become disintegrated under static cultures for 1–3 months.

**Figure 3:**
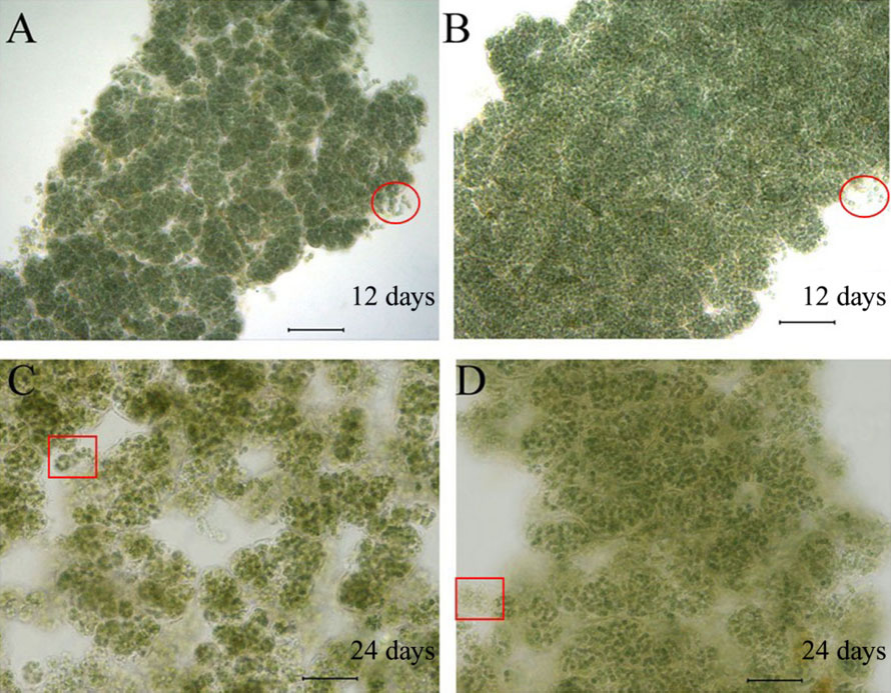
The microstructures of growing MaACs during a 24 day shake-flask culture. (A, C) The colonies respectively at 12 and 24 days under white light; (B, D) the colonies respectively at 12 and 24 days under white light plus UV-B radiation. The culture conditions were described in the legend for Fig. 2. *Circle* indicates the separating free-living cells; *square* indicates the separating tiny colonies. Bar, 5 µm.

During the shake-flask culture, the microstructures of growing MaACs were observed under microscope (Fig. 3). Colonial filaments in UV-B treated MaACs (Fig. 3B and D) appeared more tightly packed than those in non-treated MaACs (Fig. 3A and C). Also, both types of MaACs became yellowish at 24 days, as observed in MiACs that cultured under high light (Gao and Ye, 2003; Bi and Hu, 2004), which was attributed to the increased synthesis of carotenoids and also a sign of the increased oxidative damage (Salguero *et al*., 2003; Zhao *et al*., 2010). In addition, the release of free-living cells was observed in the MaACs at 12 days (Fig. 3A and B), being a way for the propagation of MaACs as observed for MiACs (Liu and Chen, 2003); differently, the propagation way for MaACs at later phase (24 days) was seemingly dependent on the release of tiny colonies (Fig. 3C and D). Although high light (e.g. 180 μmol photons m^−2^ s^−1^) and rapid agitation enhanced the biomass yield, they could also easily lead to the disintegration of filamentous colonies (Gao and Ye, 2003; Su *et al*., 2008). Thus, an equilibrium point for the macro-morphology maintenance and rapid propagation is of particular importance for massive production of MaACs. Moreover, we observed that long-term nitrogen-free culture gradually resulted in the swelling (a sign of disintegration) of MaACs. Together, large-scale production of stable MaACs could be achieved on appropriate culture conditions.

### Resistance of MaACs to desiccation stress

**Figure 4:**
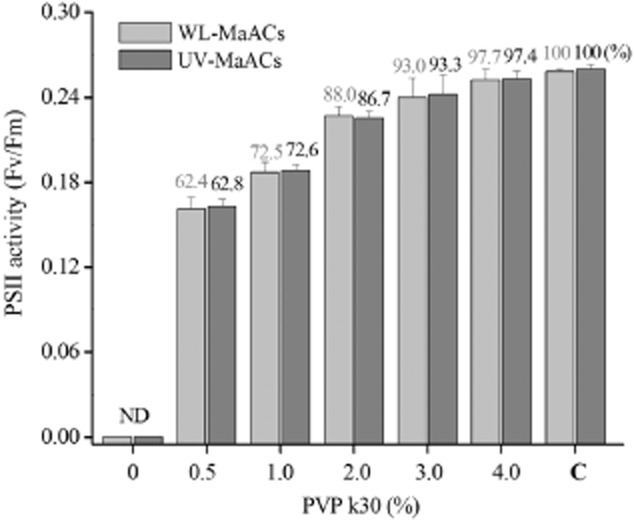
The photosystem II activity (Fv/Fm) recovery of fully desiccated MaACs that were coated with various concentrations of PVP k30 solutions. WL-MaACs and UV-MaACs, the MaACs respectively propagated under white light and UV-B treated conditions. ND, not detectable. C, the suspension of MaACs cultured under normal conditions without any desiccation treatment, as a control. Data represented as the mean ± SD (*n* = 4). PVP k30 (Sinopharm Chemical Reagent, China) was dissolved into BG11 solution to prepare various concentrations of PVP k30 solutions. The aquatic-living colonies were filtered out from the suspensions with filter paper and mixed with 4 ml PVP k30 solutions contained in plastic Petri dishes; then the prepared samples were transferred to an air-conditioned chamber (25°C, around 50% humidity) for natural air drying. The completely air-dried samples were kept in desiccation status for 3–5 days and then rehydrated by adding 4 ml sterile water into the dishes. The rehydrated samples were cultured under a PFD of 40 μmol photons m^−2^ s^−1^ for 16 h and then subjected to dark adaptation for 15 min for Fv/Fm detection as described by Qiu and colleagues (2004).

The abilities of *Nostoc* strains to withstand short- or long-term desiccation and to recover metabolic activities soon after rewetting are crucial for their success in arid environments (Dodds *et al*., 1995). The maintenance of the vitality of MaACs during desiccation stress should be crucial for their actual application. Photosystem II activity, represented by Fv/Fm (the ratio of variable to maximal chl *a* fluorescence), was typically used for evaluating the physiological recovery of *N. flagelliforme* after rehydration (Qiu *et al*., 2004). Like MiACs, the sole MaACs could not recover physiological activity after full desiccation. Polyvinylpyrrolidone (PVP) k30, a water-soluble polymer, has excellent wetting properties and readily forms films, thus making it good as a coating (Haaf *et al*., 1985; Sun *et al*., 2013). MaACs were mixed with various concentrations of PVP k30 solutions, ranging from 0.5% to 4% (W/V). Desiccation treatment of the PVP k30-coated MaACs was performed in an air-conditioned chamber. After approximately 14 h natural air drying, PVP k30-coated MaACs became fully desiccated, with approximately 11% water content, comparable with the water content of air-dried natural *N. flagelliforme*. The physiological activity in terms of Fv/Fm value was assayed after these air-dried MaACs were rehydrated for 16 h (Fig. 4). An increase of PVP k30 concentration exhibited an increased protective effect for both UV-B treated and non-treated MaACs, with 4% PVP k30 being most effective; both types of MaACs showed no significant difference in physiological recovery after desiccation stress. As a control, the Fv/Fm value was very low in rehydrated MaACs without PVP k30 coating. Also, no protective effect against desiccation stress was observed for PVP k30-coated MiACs. A macroscopic morphology or structure seemed crucial for the forming of protection by PVP k30 coating. For natural *N. flagelliforme*, EPS sheath provides a crucial protection for cells against repeated shrinking and swelling in native environments (Gao *et al*., 2012); the EPS extracted from natural *N. flagelliforme* was much more viscous than that from suspension cultures (Huang *et al*., 1998). A structural crash of less viscous EPS sheath upon desiccation stress may cause severe damages to cells. Synthetic polymers such as polyvinyl alcohol were also reported to provide desiccation protections for *Rhizobia* (Deaker *et al*., 2007); in contrast, we described in this work the protection role of PVP on macro-colonies not free-living cells or microorganisms. Thus, we propose that PVP k30 as a coating plays a potential reinforcement role for the macroscopic structures of MaACs. Generally, the solution of vitality protection for aquatic-living *N. flagelliforme* under full desiccation condition was a critical step in the actual application.

In conclusion, our results first described the development of desiccation-resistant *N. flagelliforme* MaACs for the aim of potential ecological application. Under aquatic conditions, MaACs could be massively propagated and weak UV-B treatment could result in slightly bigger MaACs. The vitality of MaACs upon full desiccation was greatly improved by coating them with 3–4% PVP k30. Compared with algal ‘seeds’ of either tiny filaments fragmented from natural *N. flagelliforme* or MiACs, PVP k30-coated MaACs were more approaching to actual application for resource restoration. The natural habitats are usually 1000–2800 m above sea level, and *N. flagelliforme* must also survive other extreme environmental conditions especially stronger visible light and UV radiation. Therefore, there still remain several improvements to be considered for the ecological application of MaACs.

## Conflict of interest

None declared.

## References

[b1] Bi YH, Hu ZY (2004). Influence of temperature, nutrients and light intensity on the growth of *Nostoc flagelliforme*. Chin J Proc Eng.

[b2] Chen LZ, Wang GH, Hong S, Liu A, Li C, Liu YD (2009). UV-B-induced oxidative damage and protective role of exopolysaccharides in desert cyanobacterium *Microcoleus vaginatus*. J Integr Plant Biol.

[b3] Dai ZJ (1992). Review on the research of *Nostoc flagelliforme*. J Ningxia Univ.

[b4] Deaker R, Roughley RJ, Kennedy IR (2007). Desiccation tolerance of *Rhizobia* when protected by synthetic polymers. Soil Biol Biochem.

[b5] Ding Z, Jia SR, Han P, Yuan N, Tan N (2013). Effects of carbon sources on growth and extracellular polysaccharide production of *Nostoc flagelliforme* under heterotrophic high-cell-density fed-batch cultures. J Appl Phycol.

[b6] Dodds WK, Gudder DA, Mollenhauer D (1995). The ecology of *Nostoc*. J Phycol.

[b7] Feng YN, Zhang ZC, Feng JL, Qiu BS (2012). Effects of UV-B radiation and periodic desiccation on the morphogenesis of the edible terrestrial cyanobacterium *Nostoc flagelliforme*. Appl Environ Microbiol.

[b8] Ferroni L, Klisch M, Pancaldi S, Häder DP (2010). Complementary UV-absorption of mycosporine-like amino acids and scytonemin is responsible for the UV-insensitivity of photosynthesis in *Nostoc flagelliforme*. Mar Drugs.

[b9] Gao KS (1998). Chinese studies on the edible blue-green alga, *Nostoc flagelliforme*: a review. J Appl Phycol.

[b10] Gao KS, Ye CP (2003). Culture of the terrestrial cyanobacterium, *Nostoc flagelliforme* (Cyanophyceae), under aquatic conditions. J Phycol.

[b12] Gao X, Ai YF, Qiu BS (2012). Drought adaptation of a terrestrial macroscopic cyanobacterium, *Nostoc flagelliforme*, in arid areas: a review. Afr J Microbiol Res.

[b13] Gao X, Yang YW, Ai YF, Luo HY, Qiu BS (2014). Quality evaluation of the edible blue-green alga *Nostoc flagelliforme* using a chlorophyll fluorescence parameter and several biochemical markers. Food Chem.

[b14] Haaf F, Sanner A, Straub F (1985). Polymers of N-vinylpyrrolidone: synthesis, characterization and uses. Polymer J.

[b15] He YY, Klisch M, Häder D (2002). Adaptation of cyanobacteria to UV-B stress correlated with oxidative stress and oxidative damage. Photochem Photobiol.

[b16] Huang Z, Liu Y, Paulsen BS, Klaveness D (1998). Studies on polysaccharides from three edible species of *Nostoc* (cyanobacteria) with different colony morphologies: comparison of monosaccharide compositions and viscosities of polysaccharides from field colonies and suspension cultures. J Phycol.

[b17] Lai YZ, Gao KS (2009). Effects of solar ultraviolet radiation on physiological characteristics of the aquatic-living colonies of *Nostoc flagelliforme* cultured indoor. Acta Hydrobiol Sin.

[b18] Liu XJ, Chen F (2003). Cell differentiation and colony alteration of an edible terrestrial cyanobacterium *Nostoc flagelliforme*, in liquid suspension cultures. Folia Microbiol.

[b19] Qian KX, Zhu HR, Chen SG (1989). The ecological conditions for *Nostoc flagelliforme* and their analysis. Acta Phytoecol Geobot Sin.

[b20] Qiu BS, Zhang AH, Liu ZL, Gao KS (2004). Studies on the photosynthesis of the terrestrial cyanobacterium *Nostoc flagelliforme* subjected to desiccation and subsequent rehydration. Phycologia.

[b21] Sa YX, Yu HF, Yang L (2013). Effect of UV-B radiation on reactive oxygen species and antioxidant enzymes of *Nostoc flagelliforme* cells. Modern Food Sci Tech.

[b22] Salguero A, de la Morena B, Vigara J, Vega JM, Vilchez C, León R (2003). Carotenoids as protective response against oxidative damage in *Dunaliella bardawil*. Biomol Eng.

[b23] Stanier RY, Kunisawa R, Mandel M, Cohen-Bazire G (1971). Purification and properties of unicellular blue-green algae (order Chroococcales). Bacter Reviews.

[b24] Su JY, He Q, Jia SR (2006). Photosynthetic and respiratory rates in liquid suspension culture cells of *Nostoc flagelliforme* Born. et Flah. Plant Physiol Comm.

[b25] Su JY, Jia SR, Chen XF, Yu HF (2008). Morphology, cell growth, and polysaccharide production of *Nostoc flagelliforme* in liquid suspension culture at different agitation rates. J Appl Phycol.

[b26] Sun H, He JT, Wang JY, Zhang SY, Liu C, Sritharan T (2013). Investigating the multiple roles of polyvinylpyrrolidone for a general methodology of oxide encapsulation. J Am Chem Soc.

[b27] Wang HY, Gong-Bao DZ, Li YM, Yan K, An LZ, Chen SY (2011). Physiological responses of *Nostoc flagelliforme* suspension cells under low-temperature stress. J Lanzhou Univ.

[b28] Ye CP, Gao KS (2004). Photosynthetic response to salt of aquatic-living colonies of the terrestrial cyanobacterium *Nostoc flagelliforme*. J Appl Phycol.

[b29] Ye CP, Zhang MC, Yang YF, Thirumaran G (2012). Photosynthetic performance in aquatic and terrestrial colonies of *Nostoc flagelliforme* (Cyanophyceae) under aquatic and aerial conditions. J Arid Environ.

[b30] Yu HF, Liu R (2013). Effect of UV-B radiation on the synthesis of UV-absorbing compounds in a terrestrial cyanobacterium, *Nostoc flagelliforme*. J Appl Phycol.

[b31] Yu HF, Jia SR, Dai YJ (2009). Growth characteristics of the cyanobacterium *Nostoc flagelliforme* in photoautotrophic, mixotrophic and heterotrophic cultivation. J Appl Phycol.

[b32] Yu HF, Jia SR, Dai YJ (2010). Accumulation of exopolysaccharides in liquid suspension culture of *Nostoc flagelliforme* cells. Appl Biochem Biotechnol.

[b33] Zhao XM, Bi YH, Hu ZY (2005). Effects of different medium on the growth and photosynthetic activity of *Nostoc flagelliforme*. J Wuhan Bot Res.

[b34] Zhao XM, Bi YH, Zhou GJ, Hu ZY (2010). The morphogenesis of *Nostoc flagelliforme* under culture conditions. Acta Hydrobiol Sin.

